# Understanding the peculiarities of the piezoelectric effect in macro-porous BaTiO_3_

**DOI:** 10.1080/14686996.2016.1245578

**Published:** 2016-11-16

**Authors:** James I. Roscow, Vitaly Yu. Topolov, Christopher R. Bowen, John Taylor, Anatoly E. Panich

**Affiliations:** ^a^ Department of Mechanical Engineering, Materials and Structures Centre, University of Bath, Bath, UK; ^b^ Department of Physics, Southern Federal University, Rostov-on-Don, Russia; ^c^ Department of Electrical and Electronic Engineering, University of Bath, Bath, UK; ^d^ Institute of High Technologies and Piezotechnics, Southern Federal University, Rostov-on-Don, Russia

**Keywords:** Piezoelectric, porous, microstructure, ferroelectric, modelling, 50 Energy Materials, 102 Porous / Nanoporous / Nanostructured materials, 107 Glass and ceramic materials, 202 Dielectrics / Piezoelectrics / Insulators

## Abstract

This work demonstrates the potential of porous BaTiO_3_ for piezoelectric sensor and energy-harvesting applications by manufacture of materials, detailed characterisation and application of new models. Ferroelectric macro-porous BaTiO_3_ ceramics for piezoelectric applications are manufactured for a range of relative densities, *α* = 0.30–0.95, using the burned out polymer spheres method. The piezoelectric activity and relevant parameters for specific applications are interpreted by developing two models: a model of a 3–0 composite and a ‘composite in composite’ model. The appropriate ranges of relative density for the application of these models to accurately predict piezoelectric properties are examined. The two models are extended to take into account the effect of 90° domain-wall mobility within ceramic grains on the piezoelectric coefficients $d_{3j}^{*}$. It is shown that porous ferroelectrics provide a novel route to form materials with large piezoelectric anisotropy $\left(d_{33}^{*}/\;\left|d_{31}^{*}\right|>>1\right)$ at 0.20 ≤ *α* ≤ 0.45 and achieve a high squared figure of merit $d_{33}^{*}$ $g_{33}^{*}$. The modelling approach allows a detailed analysis of the relationships between the properties of the monolithic and porous materials for the design of porous structures with optimum properties.

## Graphical abstract

**Figure:**
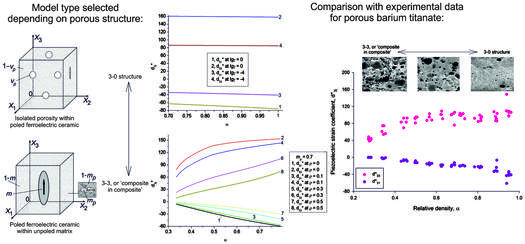

## 1. Introduction

Piezoelectric porous materials based on ferroelectric ceramics (FCs) are of interest not only due to piezoelectric, hydroacoustic and energy-harvesting characteristics,[1–4] but also as heterogeneous ferroelectric materials with intricate microgeometry–properties interrelations.[5,6] The physical properties of a porous material that exhibits electromechanical coupling due to its ferroelectric nature depends on its properties, manufacturing method, microstructure and poling conditions. Relevant aspects of the microstructure of porous ferroelectric materials include the properties of the pore-forming agents, porosity *v*_*p*_ (volume fraction of the pores in the monolithic FC), the shape and size of the pores, their orientation with respect to the poling direction, and other microgeometric features.[2,3,7,8] Among the porous materials based on the perovskite-type FCs of interest, porous lead-based PZT-type FCs (ceramic compositions based on Pb(Zr, Ti)O_3_) and composites [1–3,6–8] have been the most commonly studied in recent decades. Although the physical properties of dense monolithic BaTiO_3_ FC are well known,[9] the piezoelectric performance and related parameters of porous materials based on BaTiO_3_ [4] have yet to be studied in detail. In contrast to the numerous PZT-type FCs with complicated (heterophase) compositions near the morphotropic phase boundary, the BaTiO_3_ FC represents a monophasic material with grains split into both 180° and 90° domains.[9,10] At room temperature, the ferroelectric phase of BaTiO_3_ is tetragonal from the 4 mm symmetry class [10] and, in our opinion, the relative simplicity of the domain structure of the FC grains and the FC microstructure can enable a detailed analysis of the relationships between the properties of the monolithic and porous FC-based materials. The aim of the present paper is to interpret the piezoelectric performance of macro-porous BaTiO_3_ in a wide porosity range using both experimental and modelling approaches and consider the porous material for sensor and energy-harvesting applications.

## 2. Manufacturing and experimental data

To manufacture the porous material [4] for this study, BaTiO_3_ powder (Ferro, Stoke-on-Trent, UK) was ball-milled for 24 h with zirconia media and distilled water. A small amount of the polyethylene glycol (PEG, Sigma Aldrich, Market Harborough, UK) as a binder was added to the BaTiO_3_ powder prior to ball milling in order to facilitate uniaxial cold pressing of samples and produce crack-free green bodies. After ball-milling, the BaTiO_3_ powder was dried over night before sieving through a 150 μm mesh. Porous BaTiO_3_ samples at 0.10 ≤ *v*_*p*_ ≤ 0.72 were produced by means of the ‘burned out polymer spheres’ (BURPS) method. The porosity of all the manufactured materials was measured via the Archimedean method. In the BURPS method, ceramic powder is mixed with varying weight fractions of a volatile polymer species. In this case the volatile polymer was PEG, which burns out during the sintering stage to leave randomly distributed pores in the structure, whilst providing good control over the final porosity of the materials. In this method, the resulting pore size and morphology are similar to those of the pore-forming agent.

**Figure 1.**
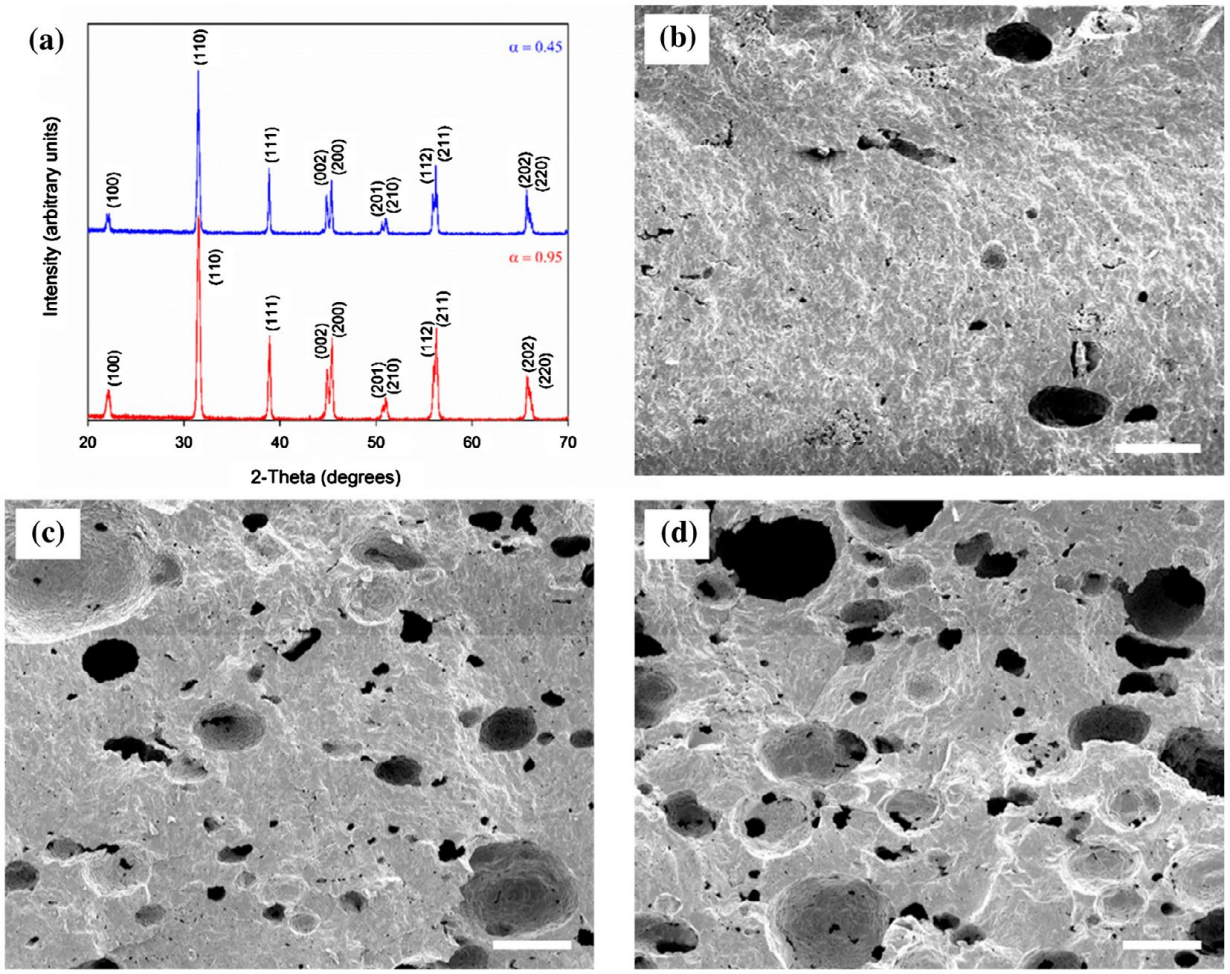
(a) XRD patterns of dense (*v*_*p*_ = 0.05) and porous (*v*_*p*_ = 0.55) BaTiO_3_ after sintering, both exhibiting fully formed perovskite crystal structure; and SEM micrographs of porous BaTiO_3_ with (b) *v*_*p*_ = 0.11, (c) *v*_*p*_ = 0.19, and (d) *v*_*p*_ = 0.28, demonstrating the increase in interconnection between macro-pores with increasing in *v*_*p*_. All scale bars are 200 μm in length.

After uniaxial pressing at 300 MPa to form pressed pellets (13 mm diameter) the samples were sintered in air in an Elite Thermal Systems Ltd (Gillingham, UK) furnace (Model No. BRF14/10–2416 CG) at 1300 °C for two hours. A two-hour dwell stage at 400 °C during initial heating was carried out to burn out the binder/pore-forming agent, and the ramp rate was ±60 °C/h. X-ray diffraction (XRD, Philips PW1730, Guildford, UK) analysis confirmed a fully perovskite structure in both the monolithic and high-porosity samples, see Figure 1(a). The pore size in samples at the lower porosity (*v*_*p*_ = 0.1–0.3) was estimated from scanning electron microscopy (SEM, JEOL JSM6480LV, Peabody, MA, USA) images (see, for instance, Figure 1(b–d)), and the average size of the macro-pores was 150 μm. The size of the macro-pores can vary from 50 to 400 μm, which is thought to be due to a combination of a variation in the size of the pore-forming agent and coalescence of pore-forming agent in high porosity samples. There is also some micro-porosity that is not thought to be caused by the pore-forming agent, and the size of such pores is approximately 3–4 μm. Both types of porosity appear to be spherical.

To ‘pole’ the porous BaTiO_3_ samples, electrical corona poling was performed in air at 115 °C with a 14 kV voltage applied from a 35 mm point source. Piezoelectric strain coefficients $d_{3j}^{*}$ (*j* = 1 and 3) were measured on the manufactured dense and porous samples by means of a Take Control Piezometer System PM25 with an adapter for measuring $d_{31}^{*}$. The dielectric properties of the same samples were studied using impedance spectroscopy via a Solartron 1260 and 1296 Dielectric Interface (Solartron Analytical, Farnborough, UK), and based on data, the relative permittivity of the stress-free sample $\varepsilon_{33}^{*\sigma }$ was evaluated in the wide porosity range.[4]

**Figure 2.**
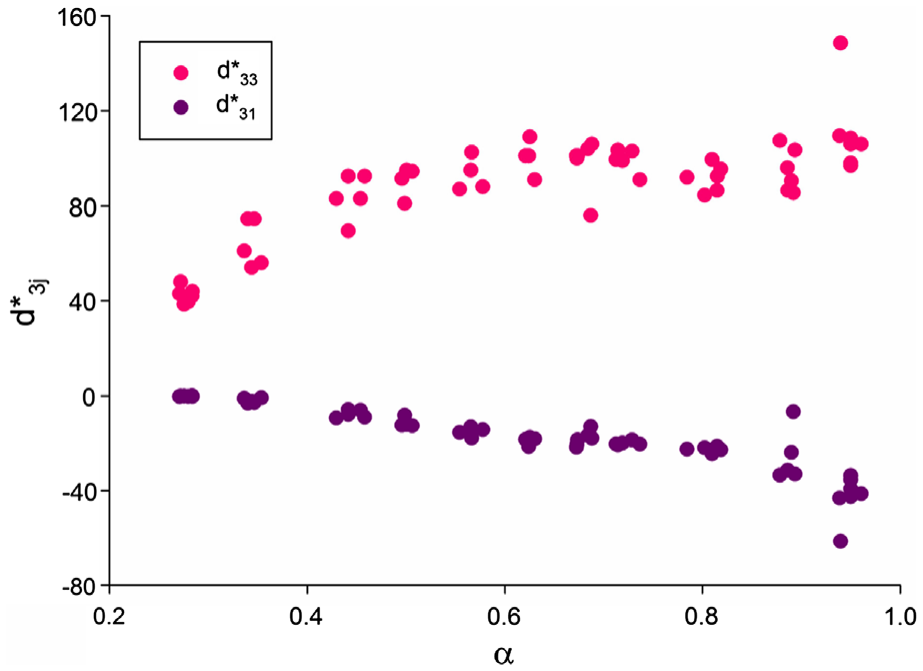
Piezoelectric coefficients $d_{3j}^{*}$ (in pC/N) measured on poled porous BaTiO_3_ samples at room temperature. *α* is relative density of the sample.

Figure 2 shows the experimental dependence of the piezoelectric coefficients $d_{3j}^{*}$ on the relative density *α* = 1 – *v*_*p*_ of the porous materials. At 0 < *α* < 1, the relative permittivity $\varepsilon_{33}^{*\sigma }$ increases monotonously, and its derivative is *d*($\varepsilon_{33}^{*\sigma }$)/*dα* ≈ 1500. Data from Figure 2 suggest that the porous structure influences the piezoelectric effect on the poling (*j* = 3) and lateral (*j* = 1) directions in different ways, and such a response is a result of the specific arrangement of pores formed in the samples. The piezoelectric anisotropy $d_{33}^{*}$/$d_{31}^{*}$ undergoes relatively small changes at higher levels of density in the range 0.50 < *α* < 1, but at lower densities in the range 0.20 ≤ *α* ≤ 0.45 a much larger degree of the piezoelectric anisotropy (i.e. $d_{33}^{*}/\;\left|d_{31}^{*}\right|>>1$) is observed. At 0.20 ≤ *α* ≤ 0.45, values of the piezoelectric coefficient $d_{33}^{*}$ (Figure 2) are comparable to $d_{33}^{*}$ of highly anisotropic PbTiO_3_-type FCs, however the relative permittivity $\varepsilon_{33}^{*\sigma }$ of the porous BaTiO_3_ samples is larger than that of the monolithic PbTiO_3_-type FCs.[10,11] In this case we see that there is an opportunity to replace lead-containing FC materials with porous BaTiO_3_ materials for piezoelectric based applications [12–14] such as sensors, acoustic receivers, active elements of non-destructive testing devices, and devices for medical diagnostics.

## 3. Interpretation and comparison of results on the piezoelectric performance

Earlier modelling studies have focused on porous ferroelectric PZT-type materials (see e.g. [1,3,5,6,15–17]) and it is clear that different manufacturing methods, as well as various pore-forming agents and microgeometric features of the porous ferroelectrics [18–21] make it difficult to use a reliable model across the whole porosity range. Recent attempts have explored composite models [6,15–17] for interpreting the piezoelectric performance of the porous PZT-type medium, and this circumstance stimulated our further analysis.

**Figure 3.**
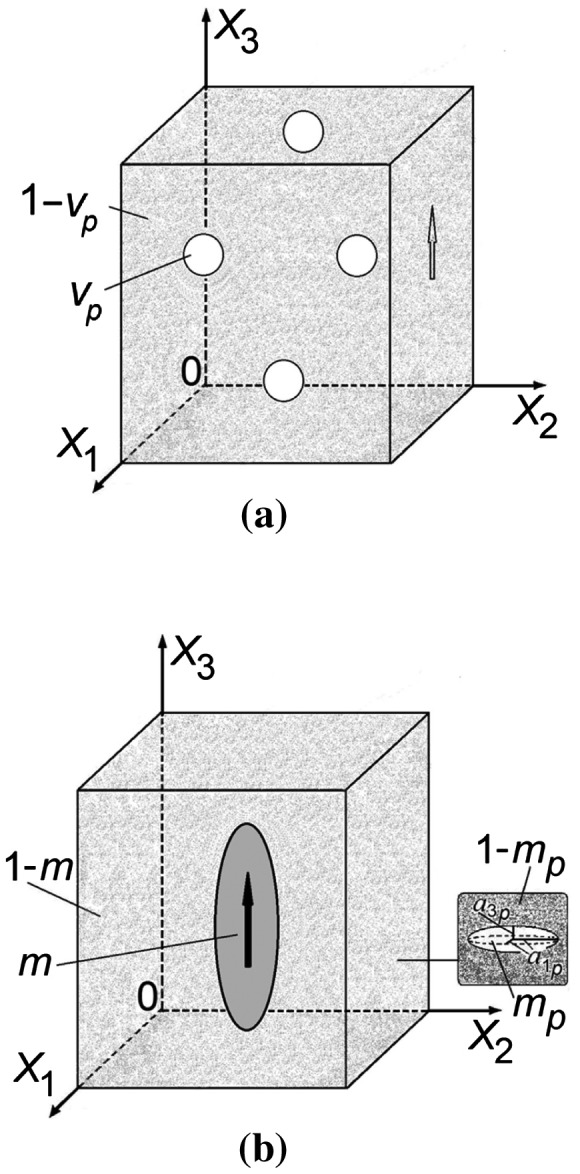
Schematics of the porous structure at (a) high relative densities (3–0 composite model) and (b) low relative densities (‘composite in composite’ model). In (a) *v*_*p*_ is the effective porosity of the sample, and its remanent polarisation vector is shown with the arrow on the right side. In (b) *m* is the volume fraction of the monolithic poled FC inclusion with semiaxes *a*_1_ = *a*_2_ and *a*_3_. 1 – *m* is the volume fraction of the unpoled porous ceramic matrix wherein *m*_*p*_ is the volume fraction of air inclusions with semiaxes *a*_1*p*_ = *a*_2*p*_ and *a*_3*p*_. The remanent polarisation vector of the FC inclusion is shown with the arrow. The effective porosity of the sample shown in (b) is *v*_*p*_ = (1 – *m*)*m*_*p*_.

To interpret the piezoelectric properties of porous BaTiO_3_, we now put forward two models of the piezo-active composite. Taking into consideration the SEM images from Figure 1(b) and 1(c), at relatively low porosity levels *v*_*p*_, we assume that the FC matrix contains isolated spherical air inclusions, and these inclusions are regularly distributed throughout the material; see Figure 3(a). Such a composite is described by 3–0 connectivity in terms of work.[3,12,13] The effective electromechanical (i.e. elastic, piezoelectric and dielectric) properties of the porous 3–0 composite are determined in the matrix form by using the dilute approach,[5] and in this case any interaction between the inclusions can be neglected. The matrix of the effective properties of the 3–0 composite is given by:

(1) $$\|C^{*}\left\|=\right\|C^{\left(1\right)}\|\left[\left\|I\right\|-\left(1-\alpha \right){\left(\left\|I\|-\alpha \|S\right\|\right)}^{-1}\right]$$

In Equation (1) || *C* ^(1)^ || is the 9 × 9 matrix that describes the electromechanical properties of the monolithic FC, ||*I*|| is the 9 × 9 identity matrix, ||*S*|| is the 9 × 9 matrix containing components of the Eshelby electroelastic tensor,[3,22] and *α* is the relative density. Elements of ||*S*|| depend [3,22] on the shape of the inclusions and on the electromechanical properties of the monolithic FC medium that surrounds the inclusions. The ||*C* ^(1)^|| matrix from Equation (1) is represented as follows:

(2) $$\|{\text{C}}^{\left(1\right)}\|=\left(\begin{array}{cc} \|c^{(1),E}\| & \|e^{\left(1\right)}{\|}^{t}\\ \|e^{\left(1\right)}\| & -\|\varepsilon^{(1),\xi }\| \end{array}\right)$$

where ||*c* ^(1),*E*^|| is the 6 × 6 matrix of elastic moduli measured at constant electric field, ||*e* ^(1)^|| is the 6 × 3 matrix of piezoelectric coefficients, and ||*ε* ^(1),*ξ*^|| is the 3 × 3 matrix of relative permittivities measured at constant mechanical strain. The superscript *t* in Equation (2) denotes the transposition. The matrix of effective electromechanical constants ||C*|| from Equation (1) has the form shown in Equation (2). Table 1 shows the room-temperature elastic moduli, piezoelectric coefficients and relative permittivities of the poled monolithic BaTiO_3_ FC used for the modelling.

**Table 1.** Room-temperature elastic moduli $c_{pq}^{E}$ (in 10^10^ Pa), piezoelectric coefficients *e*_*ij*_ (in C/m^2^) and relative permittivities $\varepsilon_{pp}^{\xi }$ of monolithic BaTiO_3_ FC.^a^[9]

$c_{11}^{E}$	$c_{12}^{E}$	$c_{13}^{E}$	$c_{33}^{E}$	$c_{44}^{E}$	*e*_31_	*e*_33_	*e*_15_	$\varepsilon_{11}^{\xi }$	$\varepsilon_{33}^{\xi }$
15.0	6.6	6.0	14.6	4.4	–4.35	17.5	11.4	1115	1260

^a^ Piezoelectric strain coefficients *d*_31_ = –78 pC/N, *d*_33_ = 190 pC/N and *d*_15_ = 260 pC/N.

As the porosity level in the material increases there is an increase in the interconnection of the pores; see, for example, Figure 1(d). In this porosity range we therefore apply a ‘composite in composite’ model as in Figure 3(b) where *m* is the volume fraction of the monolithic poled FC inclusion, 1 – *m* is the volume fraction of the unpoled porous ceramic matrix and *m*_*p*_ is the volume fraction of air inclusions. The effective porosity is therefore *v*_*p*_ = (1 – *m*)*m*_*p*_. It is assumed that the monolithic poled FC (piezoelectric) inclusions are regularly distributed in a porous non-poled FC (piezo-passive) matrix. Our approach is to ensure the non-poled (i.e. piezo-passive) material surrounding the poled FC inclusion is consistent with model concepts [23] based on the distribution of unpoled and poled regions within the FC material. The FC–air network and modelling were used to interpret the piezoelectric properties and related parameters of porous lead zirconate titanate FCs; however, the shape of poled and unpoled regions was not discussed in detail.[23]

The shape of the poled FC inclusion in our model (Figure 3(b)) is described by the equation (*x*_1_/*a*_1_)^2^ + (*x*_2_/*a*_1_)^2^ + (*x*_3_/*a*_3_)^2^ = 1 relative to the axes of the rectangular co-ordinate system (*X*_1_ *X*_2_ *X*_3_), where semiaxes of the spheroid are *a*_1_ = *a*_2_ and *a*_3_, and *ρ* = *a*_1_/*a*_3_ is the aspect ratio. In a limiting case, *ρ* = 0, the inclusion has the form of a circular cylinder. The spheroidal air inclusions are uniformly distributed in the unpoled FC matrix (see inset in Figure 3(b)), and the shape of the air inclusions are characterised by the aspect ratio *ρ*_*p*_ = *a*_1*p*_/*a*_3*p*_, where *a*_1*p* _= *a*_2*p*_ and *a*_3*p*_ are semiaxes of the air inclusion. We also assume that the radius of each air inclusion is much smaller than the length of each semiaxis *a*_*j*_ of the FC inclusion. The composite shown in Figure 3(b) is characterised by 0–3–0 connectivity at *ρ* > 0 or by 1–3–0 connectivity at *ρ* = 0.

In the present model of the ‘composite in composite’, the electromechanical interaction between the poled FC inclusions is considered. The effective properties of the composite are determined by means of the effective field method.[3,5] Following this method, we represent the ||C*|| matrix of effective properties as:

(3) $$\|C^{*}\left\|=\right\|C^{\left(2\right)}\|+m\left(\|C^{\left(1\right)}\left\|-\right\|C^{\left(2\right)}\|\right){\left[\left\|I\right\|+\left(1-m\right)\|S\|\|C^{\left(2\right)}{\|}^{-1}\left(\|C^{\left(1\right)}\left\|-\right\|C^{\left(2\right)}\|\right)\right]}^{-1}$$

In Equation (3) the matrices of the electromechanical properties ||*C* ^(1)^|| (poled FC inclusions) and ||*C* ^(2)^|| (porous FC matrix) have the form shown in Equation (2), *m* is the volume fraction of the poled FC inclusions, ||*I*|| is the identity matrix, and ||*S*|| is the matrix that contains components of the Eshelby electroelastic tensor.[3,5] The ||*C* ^(2)^|| matrix is written by analogy with the || C* || matrix from Equation (1):

(4) $$\|C^{\left(2\right)}\left\|=\right\|C^{\left(FC\right)}\|\left[\left\|I\right\|-m_{p}{\left(\left\|I\right\|-\left(1-m_{p}\right)\left\|S\right\|\right)}^{-1}\right]$$

In Equation (4) ||*C* ^(*FC*)^|| characterises the properties of the non-poled FC medium surrounding the air inclusions, and *m*_*p*_ is their volume fraction (Figure 3(b)). In this work we consider the aspect ratio of the air inclusion *ρ*_*p*_ >> 1. The presence of oblate-shaped air inclusions in the FC matrix strongly influences the lateral piezoelectric effect, and increasing the volume fraction of these inclusions *m*_*p*_ at *ρ*_*p*_ = const leads to a decrease of the |$d_{31}^{*}$| of the composite as a whole.

**Figure 4.**
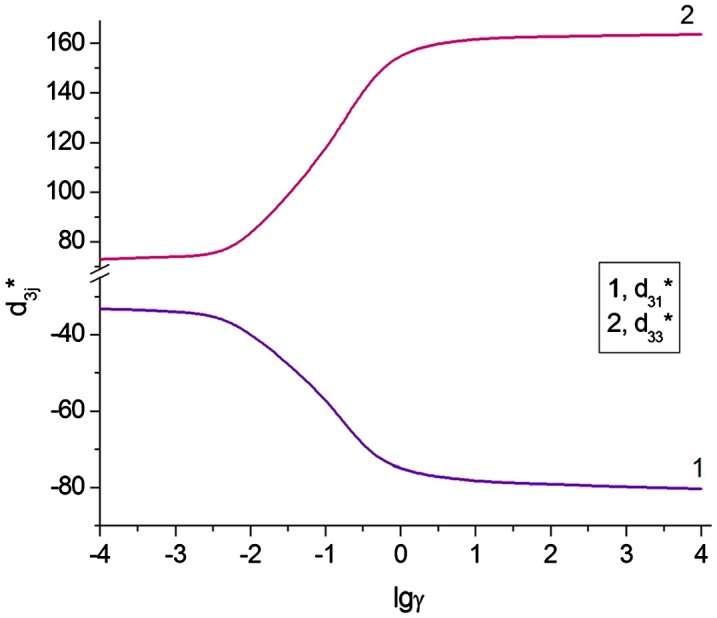
Piezoelectric coefficients $d_{3j}^{*}$ (in pC/N) of the poled monolithic BaTiO_3_ FC vs. the mobility of 90° domain walls in grains (calculations based on formulae [24]).

Absolute values of the piezoelectric coefficients $d_{3j}^{*}$ of the monolithic poled FC (if we extrapolate experimental data from Figure 2 to *α* = 1) are smaller than those from Table 1. A decrease of |$d_{3j}^{*}$| may be the result of a restricted mobility of domain walls in FC grains, and this effect was studied by Aleshin.[24] Following the model concept,[24] we assume that during poling of the FC sample, the 180° domains are removed. As a result, each grain is assumed to be split into the 90° domains that are separated by planar walls, and the 90° domain-wall displacements are caused by an external field, either electric or stress. The electromechanical properties of the monolithic poled FC depend on lg(γ) that characterises the mobility of the 90° domain walls, where γ = (*Hc*)^−1.^10^−6^ Pa, *H* is the average width of the domain, and *c* links the domain-wall displacement *x* and thermodynamic pressure *f* in accordance with the relation [24] *f* = *cx*. A dependence of the piezoelectric properties of the monolithic FC on the mobility of the 90° domain walls is graphically represented in Figure 4.

**Figure 5.**
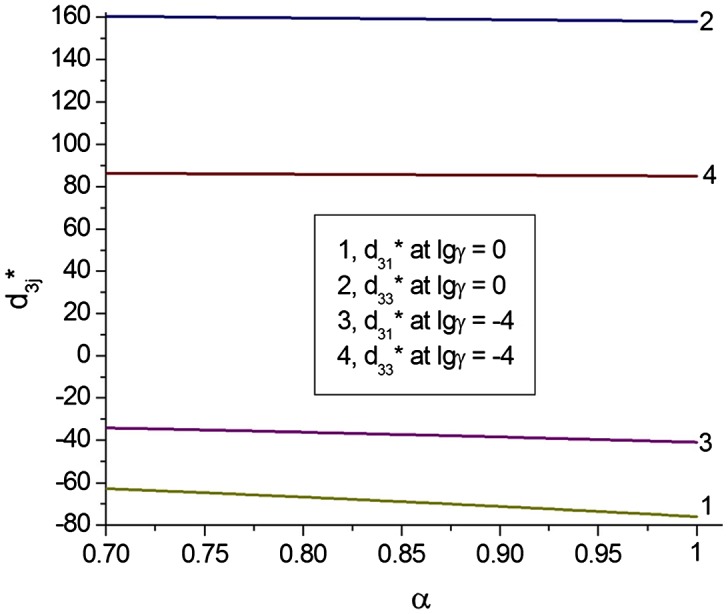
Piezoelectric coefficients $d_{3j}^{*}$ (in pC/N) of the poled porous BaTiO_3_ material (3–0 connectivity, see the model in Figure 3(a)) at the fixed mobility of 90° domain walls in FC grains. Calculations were performed using full sets of electromechanical constants [24] of the poled monolithic BaTiO_3_ FC at either lg(γ) = 0 (curves 1 and 2, moderate mobility of 90° domain walls) or lg(γ) = –4 (curves 3 and 4, low mobility of 90° domain walls).

Comparing data from Figures 2 and 5, we state that the model of the 3–0 composite shown in Figure 3(a) can be effectively applied to interpret the piezoelectric performance of the studied porous material at a relative density range of 0.7 < *α* < 1 as there is a limited change in $d_{33}^{*}$ and only a small decrease in the magnitude of $d_{31}^{*}$ with a decrease in density in this range for both model and experiment. In this case the mobility of the 90° domain walls in FC grains is characterised by –4 ≤ lg(γ) ≤ 0.

**Figure 6.**
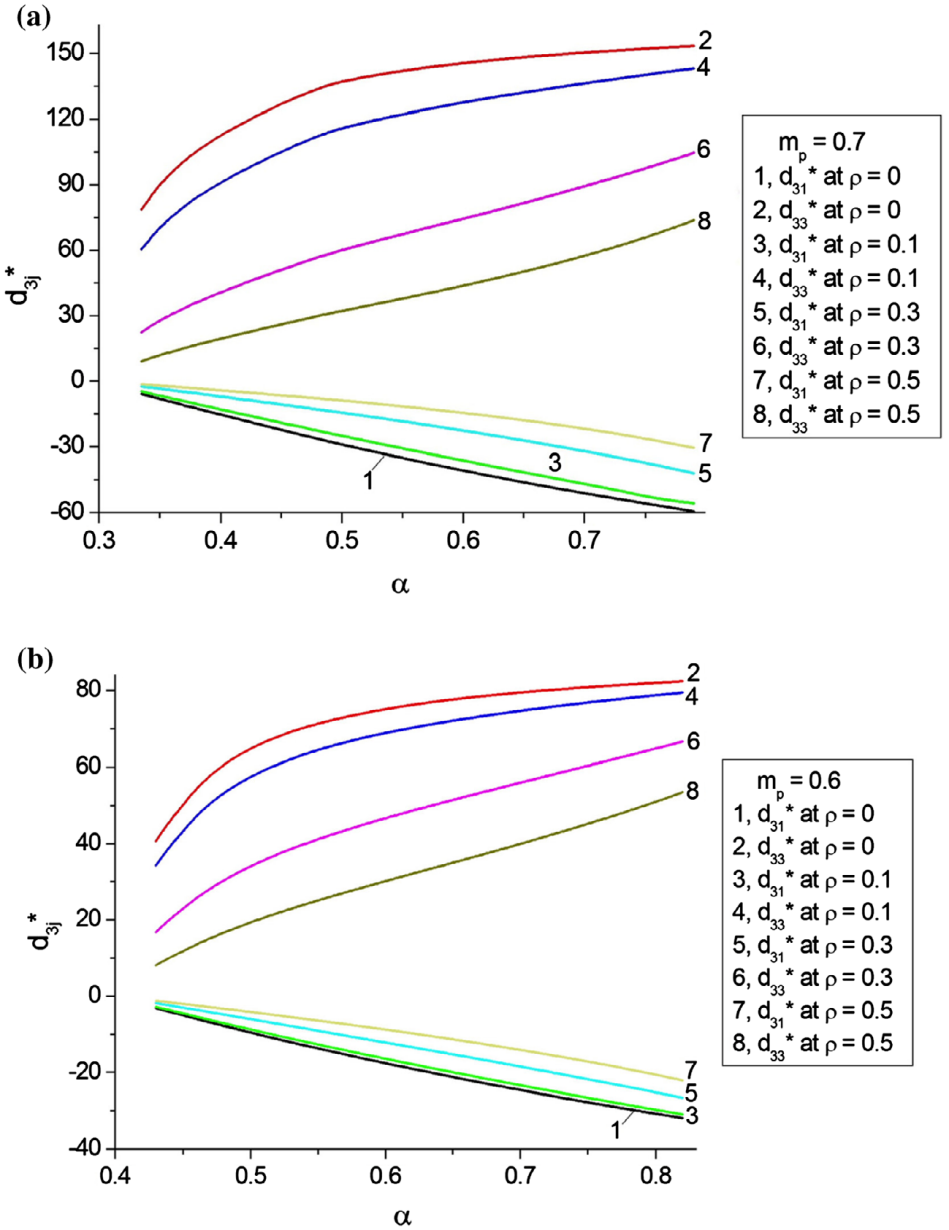
Piezoelectric coefficients $d_{3j}^{*}$ (in pC/N) of porous BaTiO_3_ at the fixed aspect ratio *ρ* of the poled FC inclusions in the porous non-poled FC matrix with oblate spheroidal air inclusions (*ρ*_*p*_ = 100, see the ‘composite in composite’ model in Figure 3(b)). The domain-wall mobility in FC grains of the poled FC inclusions is characterised by lg(γ) = 0. The volume fraction of the air inclusions in the porous matrix is either *m*_*p*_ = 0.7 (a) or *m*_*p*_ = 0.6 (b).

At lower relative densities, where *α* < 0.7, we apply the model of the ‘composite in composite’ at the porosity of the FC matrix *m*_*p*_ = 0.6–0.7, and the results of our calculations are shown in Figure 6. In the presence of the continuous cylinder-shaped (*ρ* = 0) or isolated spheroidal (*ρ* = 0.1–0.5) poled FC inclusions, the predicted piezoelectric performance (Figure 6) is in agreement with experimental data of Figure 2 at 0.3 < *α* < 0.8. It should be added that replacing the non-poled porous FC matrix with the poled porous FC matrix leads to overestimated values of $d_{3j}^{*}$ due to the considerable electromechanical coupling in the composite with two piezoelectric components.

Replacing the prolate FC inclusions with oblate ones at *ρ* >> 1 leads to a significant decrease of the piezoelectric activity of the porous sample. Our evaluations carried out within the framework of the ‘composite in composite’ model (see Figure 3(b)) at *ρ* = 10 lead to the following $d_{33}^{*}$ values: $d_{33}^{*}$ = 3.59 pC/N (*m* = 0.7 and *m*_*p*_ = 0.7), $d_{33}^{*}$ = 2.32 pC/N (*m* = 0.6 and *m*_*p*_ = 0.7), $d_{33}^{*}$ = 4.21 pC/N (*m* = 0.7 and *m*_*p*_ = 0.6), and $d_{33}^{*}$ = 2.75 pC/N (*m* = 0.6 and *m*_*p*_ = 0.6). Oblate shaped FC inclusions do not facilitate reliable poling of the sample due to the influence of depolarisation effects, and increasing the applied electric field to achieve improved poling may lead to electric breakdown of the material. In our opinion, the presence of low piezoelectric activity, or piezo-passive, interlayers, that are formed in the porous sample during manufacture leads to an additional decrease of the piezoelectric activity and domain-wall mobility in a wide *α* range.

To compare the properties of porous BaTiO_3_ with porous lead-based ferroelectric ceramics, we now compare the normalised piezoelectric coefficients of the BaTiO_3_ reported in this work with ZTS-19, which is a Pb(Zr, Ti)O_3_-based ferroelectric composition near the morphotropic phase boundary.[6,8] According to formulae:[8]

(5) $$X*\left(v_{p}\right)/\;X^{\left(1\right)}=A_{p}{\left(v_{p}^{2}+B_{p}\right)}^{-1}+C_{p}v_{p}+G_{p}\pm H_{p}$$

In Equation (5) *X* ^(1)^ is a property of the monolithic poled FC, and *A*_*p*_, *B*_*p*_, *C*_*p*_, *G*_*p*_, and *H*_*p*_ are coefficients calculated using the least-square method to characterise the porous material. These coefficients are *A*_*p*_ = –10.239, *B*_*p*_ = 2.0, *C*_*p*_ = –2.429, and *G*_*p*_ = 6.128 for *X* = *d*_31_, *A*_*p*_ = –0.206, *B*_*p*_ = 1.0, *C*_*p*_ = 0.047, and *G*_*p*_ = 1.202 for *X* = *d*_33_, and *A*_*p*_ = –5.316, *B*_*p*_ = –0.95, *C*_*p*_ = 0.625, and *G*_*p*_ = –4.531 for *X* = *g*_33_.[8] The relatively small difference between X*(*v*_*p*_) calculated using + *H*_*p*_ and X*(*v*_*p*_) calculated using at –*H*_*p*_ from Equation (5) enables us to assume *H*_*p*_ = 0 [6].

The data in Table 2 suggest that the porous BaTiO_3_ is associated with smaller ratios of $d_{3j}^{*}$/$d_{3j}^{\left(1\right)}$; however, the $g_{33}^{*}$/$g_{33}^{\left(1\right)}$ ratio is almost equal for the two porous ferroelectric materials, BaTiO_3_ and ZTS-19. This can be explained by the restricted domain-wall mobility, and therefore the lower relative permittivity, in the BaTiO_3_ FC grains in comparison to the ZTS-19 FC grains. The smaller relative permittivity $\varepsilon_{33}^{*\sigma }$ of porous BaTiO_3_ leads to larger values of the piezoelectric coefficient $g_{33}^{*}$ = $d_{33}^{*}$/$\varepsilon_{33}^{*\sigma }\varepsilon_{0}$ in comparison to ZTS-19. However, the values of $d_{33}^{*}$ of porous BaTiO_3_ are smaller than $d_{33}^{*}$ of porous ZTS-19 and lead to a decrease of $g_{33}^{*}$ for porous BaTiO_3_ at high porosity levels. As a result, the $g_{33}^{*}$/$g_{33}^{\left(1\right)}$ ratio undergoes relatively minor changes with porosity at *α* ≥ 0.7 (Table 2) for both BaTiO_3_ FC and ZTS-19 FC. The difference between the $g_{33}^{*}$/$g_{33}^{\left(1\right)}$ ratios of the two porous materials for *α* = 0.5 (see Table 2) may be a result of the microgeometric distinctions between highly porous ZTS-19 and BaTiO_3_ samples.

**Table 2.** Comparison of normalised piezoelectric coefficients X*(*v*_*p*_)/*X* ^(1)^ calculated using experimental data on poled porous materials.

*v*_*p*_	*α*	$d_{33}^{*}$/$d_{33}^{\left(1\right)}$, porous ZTS-19^a^	$d_{31}^{*}$/$d_{31}^{\left(1\right)}$, porous ZTS-19^a^	$g_{33}^{*}$/$g_{33}^{\left(1\right)}$, porous ZTS-19^a^	$d_{33}^{*}$/$d_{33}^{\left(1\right)}$, porous BaTiO_3_^b^	$d_{31}^{*}$/$d_{31}^{\left(1\right)}$, porous BaTiO_3_^b^	$g_{33}^{*}$/$g_{33}^{\left(1\right)}$, porous BaTiO_3_^b^
0.15	0.85	1.01	0.701	1.29	0.679	0.307	1.27
0.20	0.80	1.01	0.623	1.44	0.627	0.246	1.41
0.25	0.75	1.02	0.556	1.62	0.609	0.188	1.65
0.30	0.70	1.03	0.500	1.84	0.566	0.145	1.97
0.50	0.50	1.05	0.363	3.38	0.286	0.104	2.51

^a^ Calculated using Equation (5) and interpolation coefficients from [8].

^b^ Calculated using experimental data from the present study.

Of additional interest is to examine experimental data of the squared figure of merit ($Q_{33}^{*}$)^2^ = $d_{33}^{*}$ $g_{33}^{*}$ (Table 3) in comparison to the monolithic BaTiO_3_ in work.[9] The parameter ($Q_{33}^{*}$)^2^ is used [2,4,6] to characterise the signal–noise ratio on the longitudinal direction or performance for piezoelectric energy harvesting in longitudinal direction off-resonance. The data from Table 3 suggest that the larger values of the normalised squared figure of merit ($Q_{33}^{*}$)^2^/($d_{33}^{\left(1\right)}$ $g_{33}^{\left(1\right)}$) are achieved at lower relative densities (*α* ≈ 0.7) where a lower piezoelectric coefficient $d_{33}^{\left(1\right)}$ is measured, and this behaviour is particular to the porous BaTiO_3_ material presented in this work. The larger value of $d_{33}^{\left(1\right)}$ [9] leads to a larger value of $d_{33}^{\left(1\right)}$ $g_{33}^{\left(1\right)}$ of the FC and to a decrease in ($Q_{33}^{*}$)^2^/($d_{33}^{\left(1\right)}$ $g_{33}^{\left(1\right)}$). The values of ($Q_{33}^{*}$)^2^ ≈ 3 · 10^−12^ Pa^−1^ and ($Q_{33}^{*}$)^2^/($d_{33}^{\left(1\right)}$ $g_{33}^{\left(1\right)}$) ≈ 1.1–1.2 are achieved near max[($Q_{33}^{*}$)^2^] for the porous BaTiO_3_, and max[($Q_{33}^{*}$)^2^] is strongly linked with max$g_{33}^{*}$. We note for comparison that for the monolithic BaTiO_3_ FC from [9], the value of $d_{33}^{\left(1\right)}$ $g_{33}^{\left(1\right)}$ is approximately 2.4 · 10^−12^ Pa^−1^, and for monolithic PZT FC from [25], $d_{33}^{\left(1\right)}$ $g_{33}^{\left(1\right)}$ = 1.2 · 10^−12^ Pa^−1^. According to experimental results [25] on a lattice 3–3 PZT FC/epoxy composite with a regular arrangement of components, ($Q_{33}^{*}$)^2^/($d_{33}^{\left(1\right)}$ $g_{33}^{\left(1\right)}$) = 1.18 at *α* = 0.3, and ($Q_{33}^{*}$)^2^/($d_{33}^{\left(1\right)}$ $g_{33}^{\left(1\right)}$) = 4.37 at *α* = 0.5. These values of ($Q_{33}^{*}$)^2^/($d_{33}^{\left(1\right)}$ $g_{33}^{\left(1\right)}$) are larger than those related to porous BaTiO_3_ due to the higher piezoelectric activity of PZT and to the regular composite structure [25] that promotes a better poling of the composite sample.

**Table 3.** Normalised experimental squared figures of merit ($Q_{33}^{*}$)^2^/($d_{33}^{\left(1\right)}$ $g_{33}^{\left(1\right)}$) of poled porous BaTiO_3_ in comparison to the monolithic BaTiO_3_ FC.^a^

*v*_*p*_	*α*	($Q_{33}^{*}$)^2^/($d_{33}^{\left(1\right)}$$g_{33}^{\left(1\right)}$)
0.15	0.85	0.862
0.20	0.80	0.884
0.25	0.75	1.00
0.30	0.70	1.12
0.50	0.50	0.718

^a^ According to data,[9] $d_{33}^{\left(1\right)}$ $g_{33}^{\left(1\right)}$ = 2.4 ^· ^10^−12^ Pa^−1^ for monolithic BaTiO_3_ FC.

## 4. Conclusions

**Figure 7.**
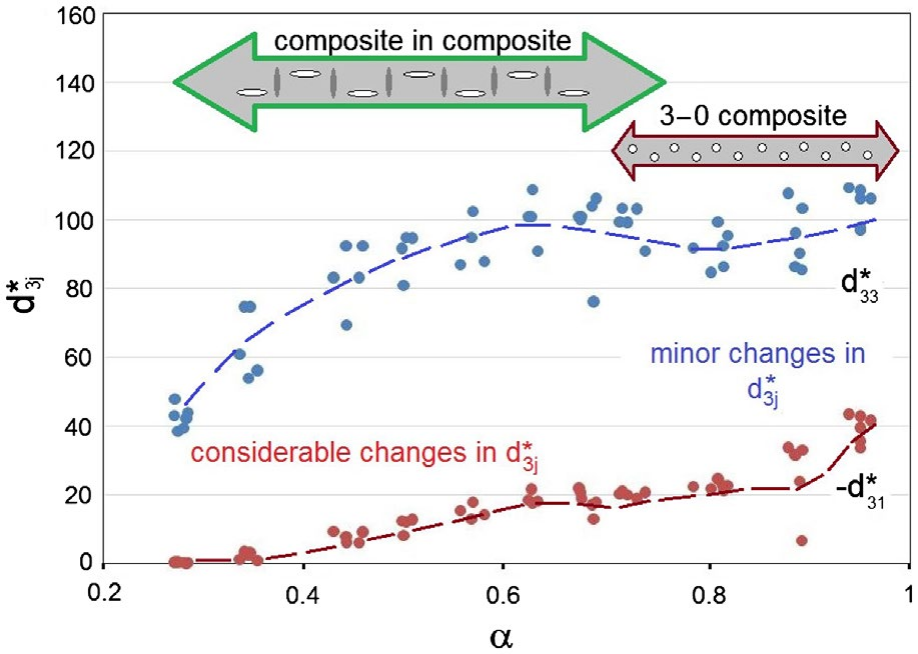
Relative density (*α*) ranges wherein models of porous BaTiO_3_ are applicable to interpret the experimental $d_{3j}^{*}$(*α*) dependence. Experimental values of the piezoelectric coefficients $d_{3j}^{*}$ in pC/N, and dotted lines are given for the benefit of the reader.

Based on both experimental and modelling methods, we have investigated in detail the piezoelectric properties of porous ferroelectric BaTiO_3_ prepared by the BURPS method. The piezoelectric performance of the prepared samples has been analysed in the wide range of the relative densities (0.3 < *α* < 1), and two models of FC–air composites have been developed; see Figure 7. At a high density level (*α* ≥ 0.7) the model of the 3–0 composite with spherical air inclusions (Figure 3(a)) accurately describes the behaviour of the piezoelectric coefficients $d_{3j}^{*}$. At a low relative density in the range 0.3 < *α* < 0.7 the ‘composite in composite’ model (Figure 3(b)) is more applicable to simulate the considerable changes in $d_{3j}^{*}$ with density for porous BaTiO_3_. It has been shown that the non-poled porous FC matrix plays an important role in the material properties, especially at forming the lateral piezoelectric response. In addition, the restricted mobility of 90° domain walls in FC grains has been taken into account in both models and the domain-wall mobility remains low (–4 ≤ lg(γ) ≤ 0 in terms of Aleshin’s work [24]) and strongly influences the piezoelectric coefficients $d_{3j}^{*}$ in the whole range of densities.

A comparison of porous lead-based ZTS-19 [6] and porous BaTiO_3_ enables us to conclude that distinctions in microgeometry of the materials and in the mobility of domain walls in their grains lead to differences in their piezoelectric properties. Such distinctions are observed in a wide relative density range for the porous media. It is shown that the properties of poled porous BaTiO_3_ are similar to those observed in porous lead-based ZTS-19 and make it a novel lead-free material for piezoelectric sensor and energy-harvesting applications; of particular note is the large piezoelectric anisotropy at 0.20 ≤ *α* ≤ 0.45. This means there is potential to exploit the longitudinal oscillation mode that is important for acoustic and transducer devices. Moreover, the models enable us to take into account the domain-wall mobility, and its changes by formation of a macro-porous structure, to be taken into account in a wide porosity range. The modelling approach allows an in-depth analysis of the relationships between the properties of the monolithic and porous materials for the design of porous ferroelectrics with optimum porosity level and geometry for transducer and piezoelectric applications.

## Disclosure statement

No potential conflict of interest was reported by the authors.

## Funding

This work was supported by Engineering and Physical Sciences Research Council; European Research Council [grant number 320963]; Souther Federal University [grant number 11.1302.2014/K].
